# Advanced automated classification and segmentation of leukemic cells using simulated optical scanning holography and active contour methods

**DOI:** 10.1117/1.JBO.30.9.096005

**Published:** 2025-09-27

**Authors:** Abdennacer El-Ouarzadi, Abdelaziz Essadike, Younes Achaoui, Abdenbi Bouzid

**Affiliations:** a Moulay Ismail University, Physical Sciences and Engineering, Faculty of Sciences, Meknès, Morocco; b Hassan First University, Higher Institute of Health Sciences, Sciences and Engineering of Biomedicals, Biophysics and Health Laboratory, Settat, Morocco

**Keywords:** leukemia, automatic classification, automatic segmentation, optical scanning holography, active contour, maximum amplitude value, phase current, accurate diagnosis

## Abstract

**Significance:**

Leukemia, a complex hematological cancer, poses significant diagnostic challenges due to the heterogeneity of leukemic cells, inter-observer variability, and lack of standardized analysis methodology. Accurate and rapid cell classification is essential to improve clinical management, optimize treatment, and reduce diagnostic errors.

**Aim:**

We propose an innovative approach combining optical scanning holography (OSH) and active contour (AC) models to automate the classification and segmentation of leukemic cells with increased accuracy.

**Approach:**

OSH is used to capture the phase current of leukocytes, providing a cost-effective, noninvasive, and simplified alternative to conventional techniques. AC models are used to improve cell segmentation. Analysis of the maximum amplitude values of the phase current allows rapid and fully automated classification.

**Results:**

The proposed approach shows a significant improvement in terms of reliability, speed, and reproducibility compared with existing methods. The integration of OSH and AC enables robust segmentation and efficient classification of leukemic cells.

**Conclusion:**

This method provides a reliable, rapid, and systematic solution for the accurate diagnosis of leukemia, enabling optimized therapeutic management.

## 1 Introduction

Leukemia is a blood cancer affecting the bone marrow and blood, leading to excessive production of abnormal white blood cells.^1^^–^^3^ These cells disrupt normal blood cell creation, causing infections, anemia, and bleeding problems. Leukemia is classified by origin (lymphoblastic or myeloid) and type (acute or chronic). Acute lymphoblastic leukemia (ALL) usually affects children, whereas acute myeloid leukemia (AML) is more common in adults.^4^ Early detection and accurate classification of leukemia subtypes are vital for treatment, requiring thorough blood and bone marrow analyses by skilled pathologists.^5^ Human error can affect this process, hence the need for a computer-aided detection and classification system. Such systems could alleviate the workload on pathologists and encourage research into automated detection of leukemia blood blasts. Most current research focuses on detecting leukemia but not on subclass classification due to their similarities. The primary classes studied include normal, ALL, AML, chronic lymphocytic leukemia (CLL), and chronic myeloid leukemia (CML). The classification of chronic leukemia is complex due to the similarity of malignant cells to normal cells, their morphological heterogeneity, and the absence of distinct markers. Current methodologies include the examination of blood smears by light microscopy, the use of flow cytometry to identify specific markers, and molecular biology approaches such as genetic sequencing. However, these methodologies have inherent limitations, including their reliance on human expertise, their high cost, and their inability to accurately capture certain subtle morphological features.

Electron microscopy, although rarely used routinely, provides superior resolution of cell structures, revealing neglected details such as chromatin texture and membrane changes. It has also been suggested that automated diagnosis of chronic leukemia could be improved by combining optical scanning holography with optical correlation techniques. This approach would improve diagnostic accuracy and reduce inter-observer variability.^6^^,^^7^

Acute leukemias are divided into myeloid and lymphoid types based on clinical, pathological, and cytological features. Myeloid leukemias are further classified into acute and chronic.^8^ Myeloid cells proliferate uncontrollably due to acquired genetic changes, affecting various stages of differentiation (from blast to granulocyte) in the bone marrow. Typically, granulocyte series cells dominate the peripheral blood smear. A diagnosis of acute myeloid leukemia occurs with over 20% of myeloid blasts present. Myeloid blasts have distinct morphological characteristics compared with other lineages.^9^ Annually, around 23,000 leukemia cases are diagnosed in the US, resulting in ∼4500 deaths, with childhood cases totaling 3000.^10^ Diagnosis relies on complete blood count (CBC) tests and microscopic examination of blood and bone marrow samples to identify and classify blood blasts, a process that requires significant training due to the risk of misidentification, which can lead to improper treatment and high mortality rates in the early days of care.^11^

Automated systems using machine learning can classify whole blood images as normal or leukemic but struggle to detect and classify atypical cells critical for diagnosis. Although there are public datasets of leukemia-presenting blood smears, they are insufficient for training automated systems due to contamination with other cells. There is no existing publicly available dataset solely containing leukemia patient blood blasts nor are datasets with only atypical cells suitable for developing effective automated detection methods.

## 2 Limits of Existing Diagnostic Methods

According to the state of the art, various methods incorporating deep learning (DL), deep neural networks (DNN), and medical image processing can be used to detect leukemic cells from blood smear and bone marrow images.^12^^,^^13^

For the diagnosis of leukemia, electron microscopic evaluation of bone marrow morphology and blood smears by experienced hematopathologists is essential. However, this procedure lacks definitive standardization and is subject to inter-observer variability.^14^ This pathology is difficult to diagnose because it is prone to human misinterpretation.^15^^,^^16^ In many cases, even an experienced pathologist may find it difficult to make a final, accurate decision. Cell counting in ALL is performed manually by pathologists and is generally subject to human error, leading to inaccurate results. This protocol is slow and subject to inter- and intra-class variation between pathologists. Pathologists can agree on leukemia diagnosis in only 76.6% of cases.^17^

A convolutional neural network (CNN)-based method for classifying normal and mature leukocytes has been proposed in other studies^18^^,^^19^ to aid in the diagnosis of leukemia. Another methodology employing CNN or artificial intelligence is utilized for the classification of normal and mature white blood cells (WBCs) to assist in the diagnosis of leukemia.^20^ The aim is to identify the morphological characteristics of WBCs and to classify them according to their type. This results in a relatively slow process that remains proportional to the databases.

## 3 Aims of the Study

The main challenges in the automated diagnosis of leukemia concern the complex characteristics of the different classes of these cancer cells, and the importance of efficient data preparation, particularly of medical images.

The proposed approach consists in developing an automatic classification and segmentation technique based on optical scanning holography combined with active contour techniques, as well as in proposing a technique based on analyzing the distribution of the values of the maximum amplitudes of the phase current of the leukemic cells in each input image, to guarantee fast and reliable classification, while ensuring rigorous evaluation to guarantee accurate and reliable results.

This work provides a complete solution for leukemic cell classification and segmentation in a clinical environment.

## 4 Materials and Methods

### 4.1 Data Acquisition and Pre-processing

The data utilized in this study are obtained from the Blood-Cell-Cancer-ALL-4-Class dataset, which is available on the Kaggle platform. This dataset was first presented in a clinical study by Hosseini et al.^21^ and contains a total of 3242 blood smear images collected from 89 patients with suspected B-cell acute lymphoblastic leukemia (B-ALL). The blood samples were stained by trained laboratory staff, and the diagnosis of cell types was confirmed by flow cytometry.

The dataset is divided into two main categories:

The benign class consists of 512 images of hematogones, a type of hematopoietic progenitor cell that morphologically resembles lymphoblasts but is not leukemic and does not require chemotherapy.

The malignant class contains 2730 images, which are divided into three subclasses representing different stages of B-ALL progression:

- Malignant-Early (Early Pre-B ALL): 979 images,
- Malignant-Pre (Pre-B ALL): 955 images,
- Malignant-Pro (Pro-B ALL): 796 images.

A series of pre-processing steps were implemented to optimize image preparation and facilitate classification based on the analysis of the maximum amplitude values of the phase current of leukemic cells and the subsequent automatic segmentation of these cells. These steps ensure reliable classification and accurate segmentation:

In accordance with the pre-processing techniques initially described by Hosseini et al.,^21^ the image set was resized to a size of 224×224  pixels to normalize the input dimensions and ensure compatibility with the analysis methods. Pixel values were then normalized between 0 and 1 to reduce variations in light intensity and improve the consistency of the results.

The images were then transformed from RGB space to HSV space, allowing the visual characteristics of the cells to be isolated with greater accuracy. This conversion facilitates the separation of the hue, saturation, and brightness components, which are essential for detailed analysis.

Following the initial pre-processing stage, the maximum amplitude values of the phase current of the leukemic cells were utilized as the primary criterion for distinguishing these cells from other features present in the images. This preliminary step effectively directs the process toward the regions of interest prior to segmentation.

In this study, the optical scanning holography (OSH) system was digitally modelled using two-dimensional (2D) images extracted from the above database. Each image represents a planar object. The holographic process is modelled using a Fourier transform, phase filtering, and inverse reconstruction to extract a simulated phase. The simulated phase is used for segmentation and classification. This approach differs from the conventional OSH method in that it is based exclusively on mathematical modelling of the optical scan, carried out line by line using a cylindrical lens.

Our proposed optical scanning holography-based system records not only the intensity of the light but also its phase. Thus, this configuration provides an output showing the distribution of the in-phase and quadrature components of the heterodyne current. However, in our study, we focus only on the phase component to extract the phase current. OSH has proven to be a particularly effective analytical technique for studying the morphological characteristics of leukemic cells.^22^ It can be used to assess dimensional parameters such as cell size, shape, and texture, and it can also detect cancer cells by identifying the initial contour using the peak of the phase current, as demonstrated in our previous works.^23^ These data, which provide remarkable precision on the internal structure of cells, are essential for the differential diagnosis between healthy and malignant cells.

In this study, the integration of optical scanning holography and active contouring techniques (which require manual determination of the initial contour) enabled fully automated segmentation, as demonstrated in one of our previous studies.^24^ In OSH, an initial contour is automatically established based on the phase current peak, whereas active contours refine these boundaries by following the morphological variations of the cells. This synergistic approach significantly reduces errors due to noise or blurred edges, thereby improving segmentation accuracy. These pre-processing steps have enabled us to build a high-quality dataset, which is essential to ensure the robustness and generalizability of predictions based on advanced morphological and amplitude analysis techniques.

### 4.2 Leukemia Classification Using Simulated Optical Scanning Holography

**Fig. 1:**
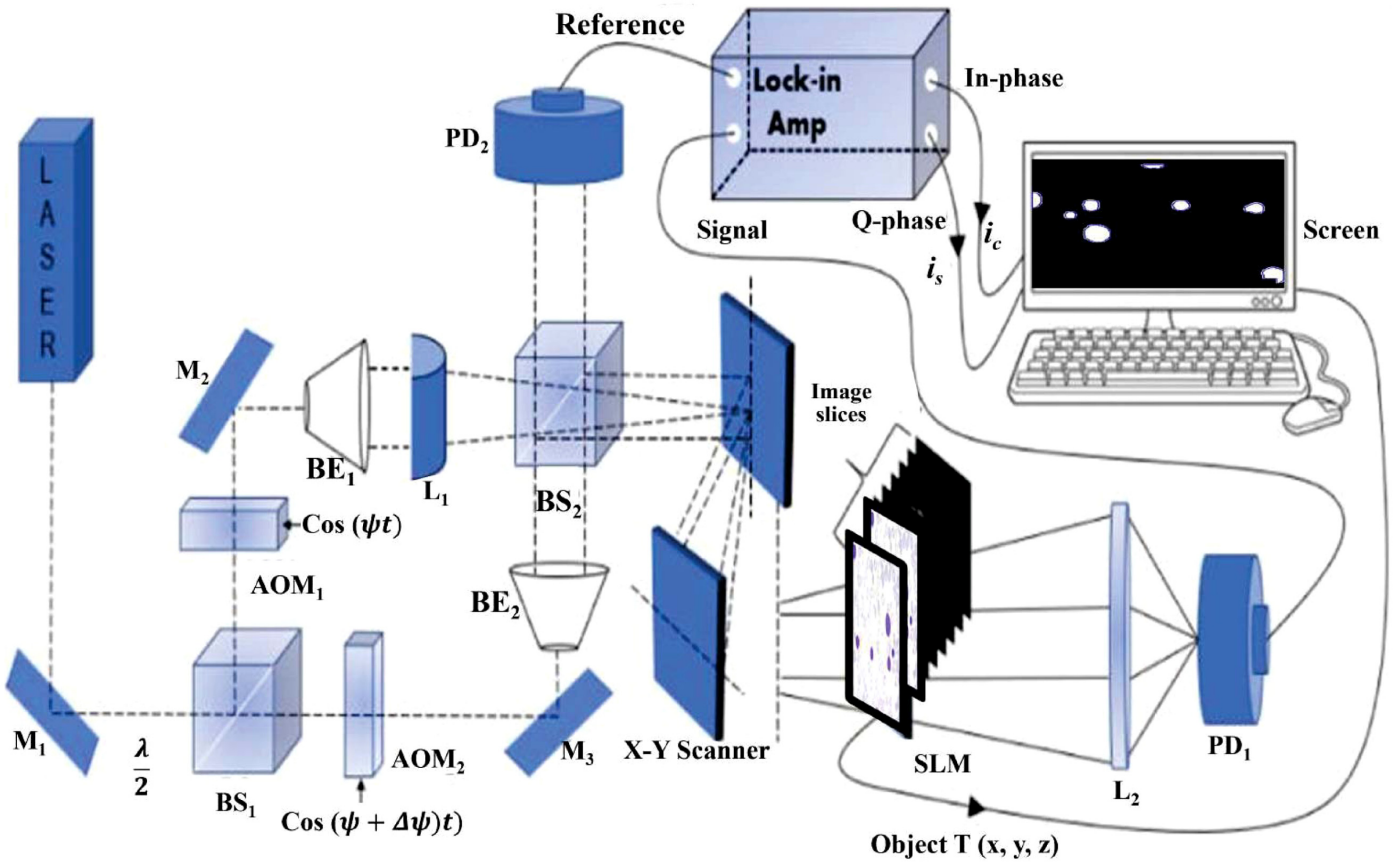
Schematic setup of the optical scanning holography (OSH) system.

Our optical system,^25^ shown in Fig. 1, is based on a Mach–Zehnder interferometer with an integrated cylindrical lens. Our figure does not refer to an existing experimental setup, but to a simulated conceptual diagram. Although our study is based solely on 2D images, we have included this theoretical configuration to clarify the physical context, facilitate understanding of the simulation results, and demonstrate the practical applicability of our approach to future experiments in accordance with academic optics standards. This principle is based on scanning the object line by line using the X to Y scanner; each line corresponds to a line of the hologram at the same vertical position. The cylindrical lens comes into play at this stage by focusing each 2D slice along a single axis, transforming the point focus into a linear focus and allowing the data to be processed in the form of reduced matrices (N,1). Each matrix thus obtained gives rise to a partial hologram, the assembly of which would lead to a stereoscopic 3D reconstruction faithful to the initial structure. Along each scan line, photo-detectors PD1 and PD2 are used to capture the optical signal scattered by the object and the heterodyne frequency information ΔΩ as a reference signal, respectively, and convert them into electrical signals for the lock-in amplifier. The in-phase and quadrature-phase outputs of the lock-in amplifier circuit produce a sine hologram, $H_{\sin}(x,y)$, and a cosine hologram, $H_{\cos}(x,y)$, to achieve a complete 2D scan of the object, as shown below

$$H(x,y)=H_{\cos}(x,y)+jH_{\sin}(x,y)=\sum_{k=0}^{N-1}H_{k}(x,y;z_{k}),$$ (1)

with

$$i_{c}=\int \{|T(x,y;z){|}^{2}*\frac{k_{0}}{2\pi z}\;\sin[\frac{k_{0}(x^{2}+y^{2})}{2z}]\}dz=H_{\sin(x,y)},$$ (2)

$$i_{s}=\int \{|T(x,y;z){|}^{2}*\frac{k_{0}}{2\pi z}\;\cos[\frac{k_{0}(x^{2}+y^{2})}{2z}]\}dz=H_{\cos(x,y)}.$$ (3)

This configuration allows the input image to be scanned line by line, optimizing data acquisition. At the output, the system produces a distribution representing the in-phase and quadrature components of the heterodyne current.

In this study, we focus only on the phase component. By applying a suitable filter, we extract the phase current after scanning the images line by line. The filter chosen in our adapted case is the pure phase filter (PPF), which is a type of correlation filter used mainly in object recognition systems. The PPF acts only on the phase of the optical wave and not on its amplitude. It is a modified version of the classic matched filter (CMF), where only the phase of the reference is retained. The expression of the filter is given by

$$H_{\mathrm{POF}}(u,v)=\frac{R^{*}(u,v)}{\left|R^{*}(u,v)\right|},$$ (4)

where $R^{*}(u,v)$ is the conjugate Fourier transform of the object to be recognized.

The maximum amplitude of the phase current in each image distribution derived from this component, known as “phase peaks,” plays a key role in two areas:

- Determination of the initial contour: these peaks are used to identify the initial contour of the leukemic cells.
- Cell classification: by measuring the maximum amplitude of the phase current, it is possible to classify leukemic cells according to their type.

This approach uses the precision of optical modelling to provide essential data for cell segmentation and classification, contributing to more accurate and automated diagnosis.

In this study, an innovative method for classifying images from blood smear microscopy is proposed, with the aim of facilitating the diagnosis and monitoring of leukemia. The proposed method is based on the analysis of the maximum amplitudes of the phase current of leukemic cells, thereby enabling effective classification. In addition, our parallel approach employs a combination of digital holography and active contour algorithms to automatically segment targeted cells.^26^ Although active contours are renowned for their segmentation efficiency, they typically necessitate manual detection of the initial contour. Our combined approach has overcome this challenge, which enables automatic and accurate determination of initial contours. This technological advancement enhances the robustness and automation of the segmentation process.

In our case study, leukemic cells can be divided into four categories based on their progression and aggressiveness: benign, malignant-early, malignant-pre, and malignant-pro.

**Fig. 2:**
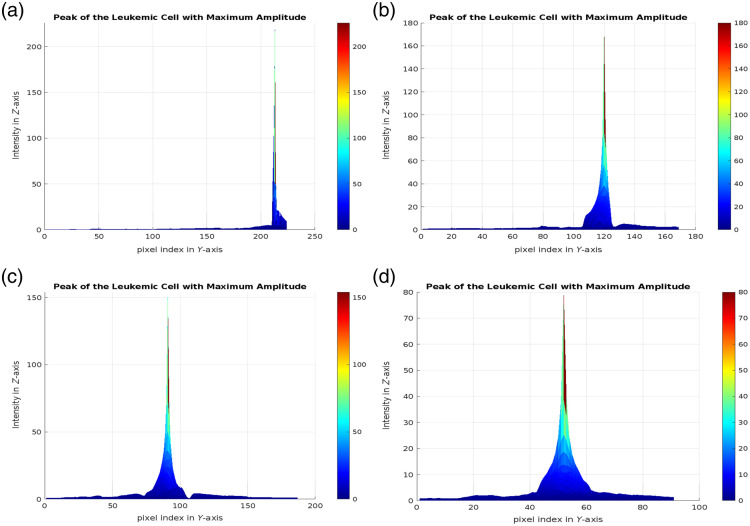
OSH-based phase component peaks distribution: (a) benign cell, (b) malignant-early cell, (c) malignant-pre cell, and (d) malignant-pro cell.

The method is based on the analysis of the maximum amplitude values in each segmented cell. For each class, 100 blood smear images were examined, giving a total of 400 images analyzed. The main steps of the approach are as follows:

- Maximum amplitude extraction: Maximum amplitude values are calculated for each cell; Fig. 2 is an example. These amplitudes reflect the structural and compositional variations of the cells that are characteristic of different stages of leukemia.
- Statistical analysis: The distributions of the maximum amplitude values are plotted for each class. The mean of the maximum amplitude values and characteristic intervals is determined for each class.
- Classification: The cells are classified according to their range of maximum amplitude, with each class corresponding to a specific range; their values are presented in Sec. 5.

**Fig. 3:**
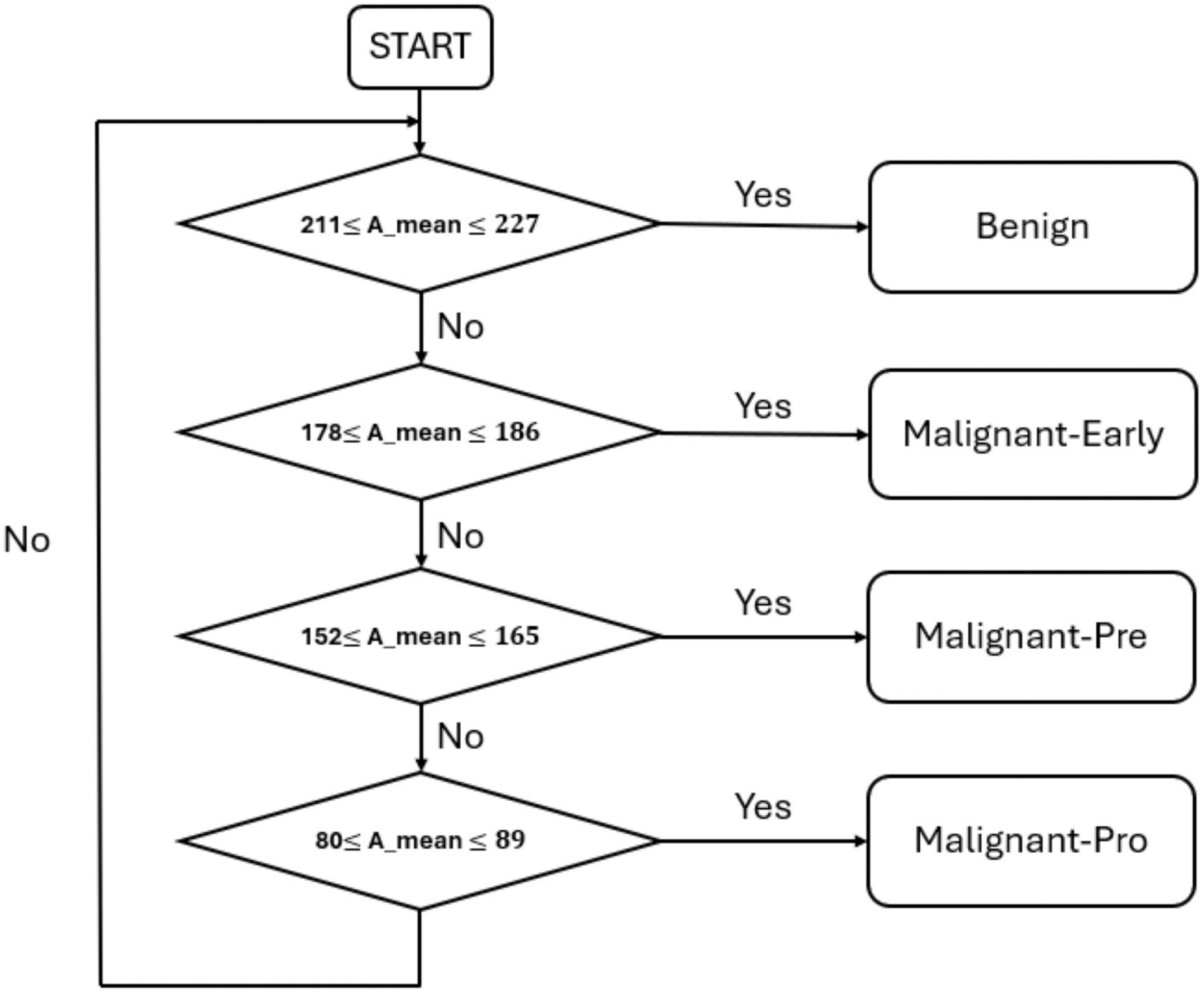
Decision tree classifying samples according to their $A_{\text{mean}}$ value.

A decision tree was created, as shown in Fig. 3, to classify the samples according to the $A_{\text{mean}}$ intervals defined for each class. The algorithm follows a hierarchical approach in which each node of the tree applies a separation condition to assign the samples to a specific class. This hierarchical approach clarifies the differentiation criteria between classes.

### 4.3 Segmentation of Classified Leukemic Cells

#### 4.3.1 Cell detection using optical scanning holography

Holography is an innovative imaging technique that, unlike traditional stereoscopic imaging techniques, has the enormous advantage of recording the three-dimensional information of an object on a single two-dimensional medium.^26^ It, therefore, provides access to the size, shape, and position of the object studied. The technique combines interference and diffraction to capture the amplitude and depth of a three-dimensional object.^27^ Interference encodes information about the object, whereas diffraction reconstructs a wave that appears to emanate from the illuminated object.^28^ This method can produce detailed and accurate images, allowing complex structures to be visualized noninvasively.^27^^,^^28^

**Fig. 4:**
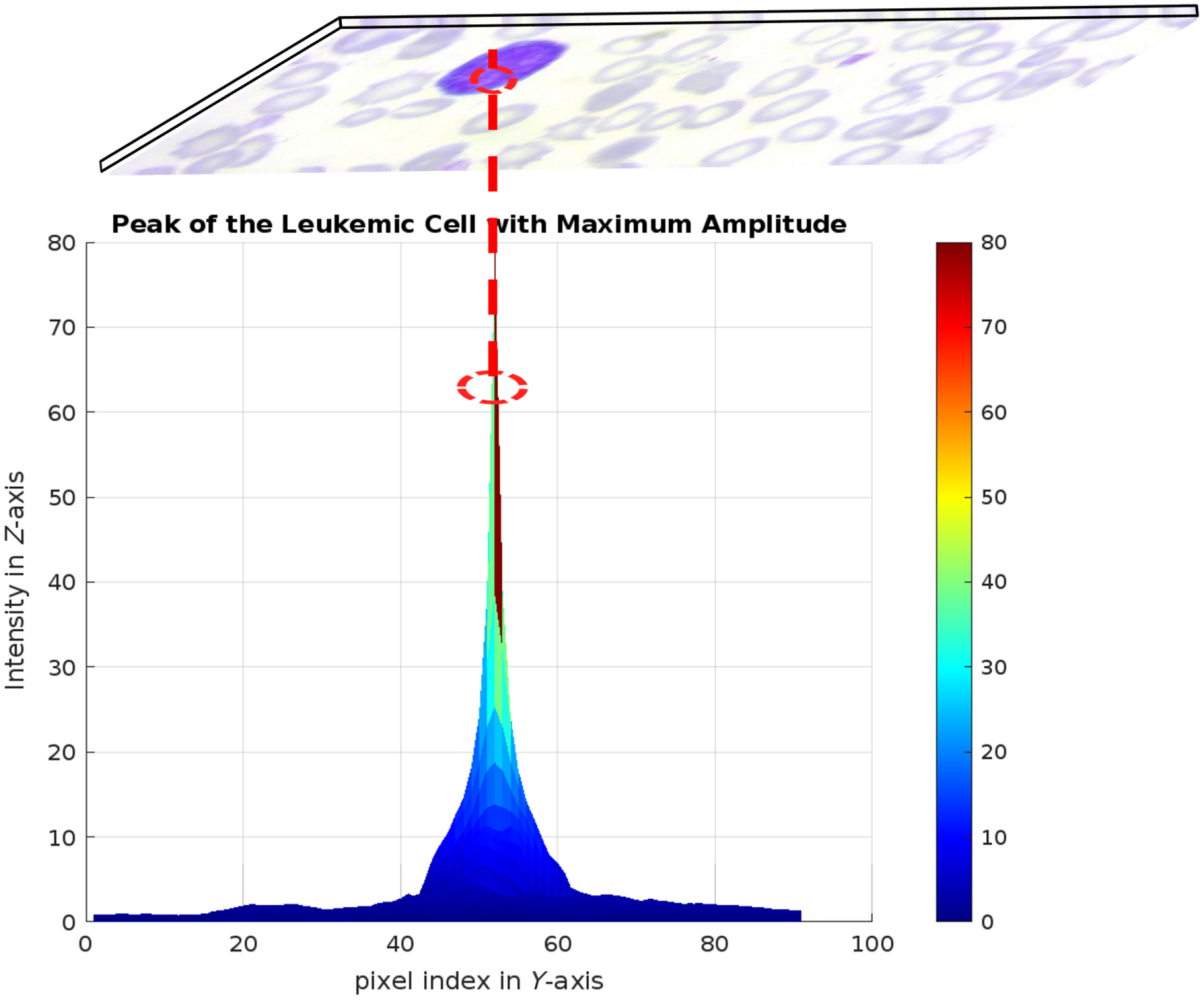
Preliminary extraction of the initial contour inside the leukemic cell by OSH-based phase component peaks.

The results of the optical process of the OSH are digitally implemented to extract the following parameters:

- C: the center of the leukemic cell.
- L: the amplitude of the peak of the phase component.
- $C_{i}$: the initial contour formed according to the principle shown in Fig. 4.

The holographic approach used in this study starts by generating a binary image of the cells, representing the potentially interesting areas in the image. The application of OSH enables phase information to be extracted. Compared with bright-field images alone, this information offers improved contrast and better detection of cellular structures. Although other techniques can be used to generate binary images, OSH adds value by improving the segmentation and identification of areas of interest with greater precision. This binary image is then transformed into the frequency domain using the 2D Fourier transform described by the following equation:

$$H(u,v)=\iint_{-\infty }^{+\infty }h(x,y)\cdot e^{-j2\pi (ux+vy)}dx\;dy,$$ (5)

where h(u,v) is the input image (in greyscale) and H(u,v) is its frequency representation. The transform is used to model a digital hologram by separating the complex spatial components of the image.

An inverse Fourier transform is applied to reconstruct the hologram:

$$h(x,y)=\iint_{-\infty }^{+\infty }H(u,v)\cdot e^{j2\pi (ux+vy)}du\;dv.$$ (6)

This process recovers amplitude and phase information from cell structures, which is then normalized to show variations in cell characteristics. The result is visualized in 3D using a data grid, where variations in amplitude values represent differences in the structure or chemical composition of the cells. This technique offers a significant advantage in the analysis of pathological cells, such as those affected by leukemia. Variations in amplitude within the hologram can reveal subtle details such as irregularities in the cell membrane or changes in intracellular composition. Cell detection using our approach is an automated and reliable method for identifying tumor cells. The process automatically generates an accurate initial contour that serves as a starting point for segmentation based on active contour algorithms.

#### 4.3.2 Active contour method

Cell segmentation is a key step in biomedical image analysis. The active contour algorithm, often called “snakes,” used in our case study is based on an energy approach. This algorithm extracts the contours of complex objects by minimizing a defined energy function.^29^^,^^30^ Using the Chan-Vese version of active contours, which is particularly suited to objects that are homogeneous in intensity, the contours are adjusted to minimize segmentation error, allowing better separation of adjacent cells.^31^^,^^32^

The algorithm starts by initializing a closed contour around the region of interest. This contour, determined by the OSH, is represented by a set of points $\left(x_{i},y_{i}\right)$ that evolve under the action of forces defined by an energy function. This function has three main components:

Internal energy ($E_{\mathrm{int}}$):

This term controls the regularity of the contour by imposing continuity and smoothing constraints. It is defined as follows:

$$E_{\mathrm{int}}=\alpha |\frac{\partial v}{\partial s}{|}^{2}+\beta |\frac{\partial^{2}v}{\partial s^{2}}{|}^{2},$$ (7)

where α and β are adjustable weights to compensate for the tension $\left(\frac{\partial v}{\partial s}\right)$ and curvature ($\frac{\partial^{2}v}{\partial s^{2}}$).

Image-based energy ($E_{\text{image}}$):

This term draws the contour toward the edges detected in the image. It is defined as the gradient of the image intensity:

$$E_{\text{image}}=-|\nabla I(x,y)|,$$ (8)

where I(x,y) is the intensity at the point (x,y).

External energy ($E_{\mathrm{ext}}$):

This term includes additional constraints or external forces to guide the contour to certain regions.

The optimization process of the algorithm is based on minimizing the sum of these energies. This is achieved by iteratively updating the contour according to the following dynamic equation:

$$\frac{\partial v}{\partial t}=-\frac{\partial E}{\partial v},$$ (9)

where v represents the contour points and E is the total energy function.

A major advantage of this algorithm is its ability to adapt to complex cell shapes, even in the presence of noise or weak intensity gradients. By adjusting the parameters α,β, and external forces, accurate cell segmentation can be achieved.

### 4.4 Limits of Contour Active Method

The active contour model is an image segmentation method that improves robustness and noise resistance over previous techniques. This semi-automatic approach requires manual initialization of a starting contour, which can be a drawback for applications requiring full automation. Another crucial addition is the use of an active contour model (ACM) for segmentation. ACMs are effective at delineating complex shapes and contours, making them well-suited to identifying regions of abnormal tissue.^33^ The active contour convergence process consists of several steps:

Initialization: An input contour is manually defined by the user, targeting the region of interest.

Dynamic evolution: The contour evolves progressively to fit the boundary of the object of interest.

Energy minimization: Convergence is achieved when the total energy is minimized.

This energy is made up of two elements:

External energy: Draws the contour toward the edges of the object.

Internal energy: Keeps the contour smooth and regular.

The total energy E can be expressed by the following equation:

$$E=E_{\mathrm{int}}+E_{\mathrm{ext}},$$ (10)

where $E_{\mathrm{int}}$ is often modelled by terms that penalise contour irregularities. $E_{\mathrm{ext}}$ is calculated from the intensity gradients of the image, attracting the contour toward areas of strong variation.

The main contributions of our method are:

- automation of active contours using holographic scanning for precise detection of the initial contour
- classification of the different types of leukemia by calculating the maximum amplitudes of the phase currents.

The current techniques used in oncology centers require the mobilization of several practitioners for the delineation of tumors,^34^^,^^35^ particularly brain tumors, on hundreds of images from the dosimetry scanner to prepare an appropriate treatment. This process can take several hours or even several days. However, thanks to our innovation based on optical scanning holography combined with optical correlation, this time can be reduced to just a few minutes. This optimizes clinical workflow and treatment accuracy.

## 5 Results and Discussion

**Fig. 5:**
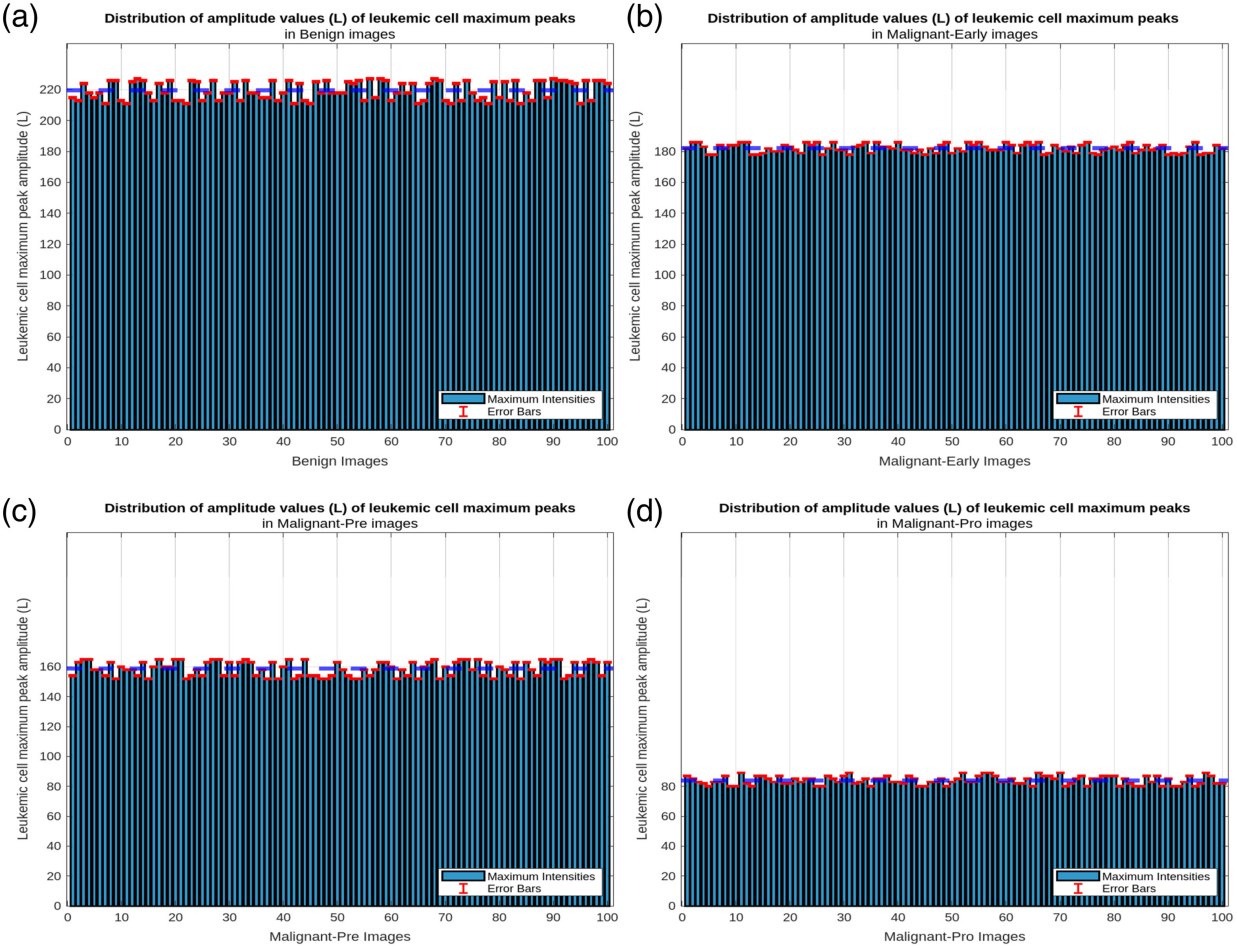
Leukemic cells, maximum amplitude distribution: (a) benign images, (b) malignant-early images, (c) malignant-pre images, and (d) malignant-pro images.

Our method calculates the amplitude values of all the cells and determines the maximum value in each input image. We then calculate the average of these maximum values from several images. This average allows us to extract a characteristic value for the four types of leukemic cells, which facilitates their classification. Figure 5 shows the distribution of the amplitude parameter for each class.

Based on the average maximum amplitudes calculated, it is possible to classify the leukemic cells into four broad classes. This is illustrated in the plots in Fig. 5, which show the mean amplitudes for each class. This figure illustrates the ability of our approach to distinguish the different classes (benign, malignant-early, malignant-pre, malignant-pro) of leukemic cells based on the calculated mean maximum amplitudes. The analysis showed that the cells in each class had characteristic maximum amplitudes, reflecting their structural and morphological differences. The distributions obtained for each class were used to define the following intervals:

- benign: $A_{\text{mean}}\in [211;227]$;
- malignant-early: $A_{\text{mean}}\in [178;186]$;
- malignant-pre: $A_{\text{mean}}\in [152;165]$;
- malignant-pro: $A_{\text{mean}}\in [80;89]$.

**Fig. 6:**
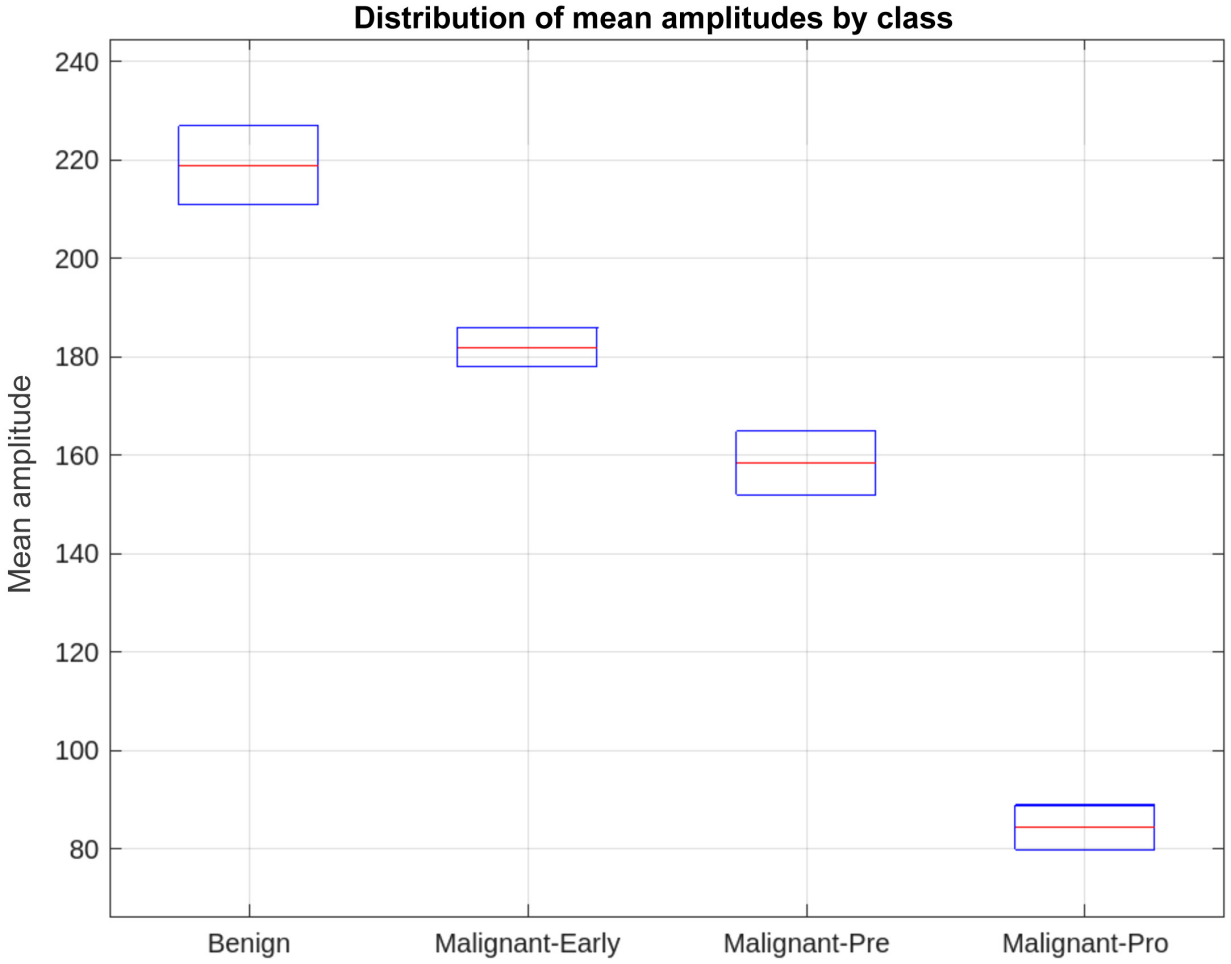
Boxplot classes.

Figure 6 of the boxplot shows the distribution of mean amplitudes for the four classes of images: benign, malignant-early, malignant-pre, and malignant-pro. Each box illustrates the dispersion of values across the first and third quartiles (Q1 and Q3), whereas the median, indicated by a line inside the box, represents the central tendency of each class. The error bars (whiskers) delimit the range of the data without taking outliers into account. Analysis of the boxplot shows a progressive decrease in mean amplitudes as we move from the benign class to the malignant-pro class, which corroborates the relevance of this feature for class differentiation. In addition, the error bars are relatively small because the data has been filtered, which limits intra-class variability and reinforces the robustness of this measure for classification purposes.

These results show that classification based on maximum amplitudes is both fast and accurate, allowing leukemic cells to be differentiated according to their stage of progression. In addition, this method has several advantages over traditional approaches:

Simplicity and speed: Unlike methods based on deep learning, this approach does not require a training phase on massive datasets or significant computational resources.

Reproducibility: As the maximum amplitudes are calculated directly from the segmented images, the results are easily verifiable.

Separation of distributions: This clear separation and nonoverlapping of values ensure more reliable classification, limiting errors and improving accuracy while making it easier to interpret the results.

Less data dependency: The method does not require large databases of annotated images, which is often a major limitation in clinical settings.

The classification of leukemic cells based on the analysis of maximum amplitudes in blood smear images is an innovative and effective method. This approach significantly reduces analysis time while maintaining high accuracy. This method could be integrated into rapid diagnostic systems and provide a viable alternative to existing advanced classification techniques.

In this study, we analyzed the distribution of maximum cell phase current amplitudes from blood smear images to classify four types of leukemic cells: benign, early malignant, pre-advanced malignant, and progressive malignant. The methodological objective of this study is to validate the feasibility of this combined approach. To do this, a small but representative sample proved sufficient. Each class was represented by a set of 100 images, as shown in Table 1, for which the maximum amplitudes were calculated, allowing a quantitative assessment of the cellular characteristics.

**Table 1:** B-ALL’s local dataset and its subclasses.

Classes	Samples	Image dimensions
Benign	100	224 × 224
Malignant-early	100	224 × 224
Malignant-pre	100	224 × 224
Malignant-pro	100	224 × 224

**Fig. 7:**
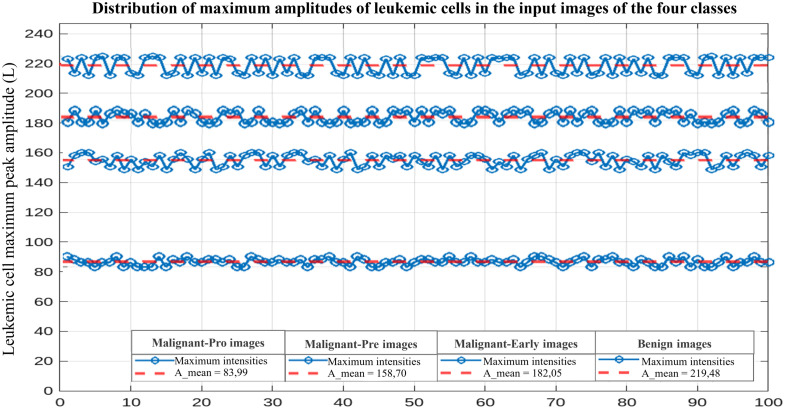
Maximum peak amplitude distribution of leukemic cells phase current in the input images of the four classes.

Figure 7 illustrates the distribution of maximum amplitudes of leukemic cells in the input images of the four classes studied, grouped together on the same figure. The nonoverlap of the distributions demonstrates the accuracy of the classification obtained by our method.

**Fig. 8:**
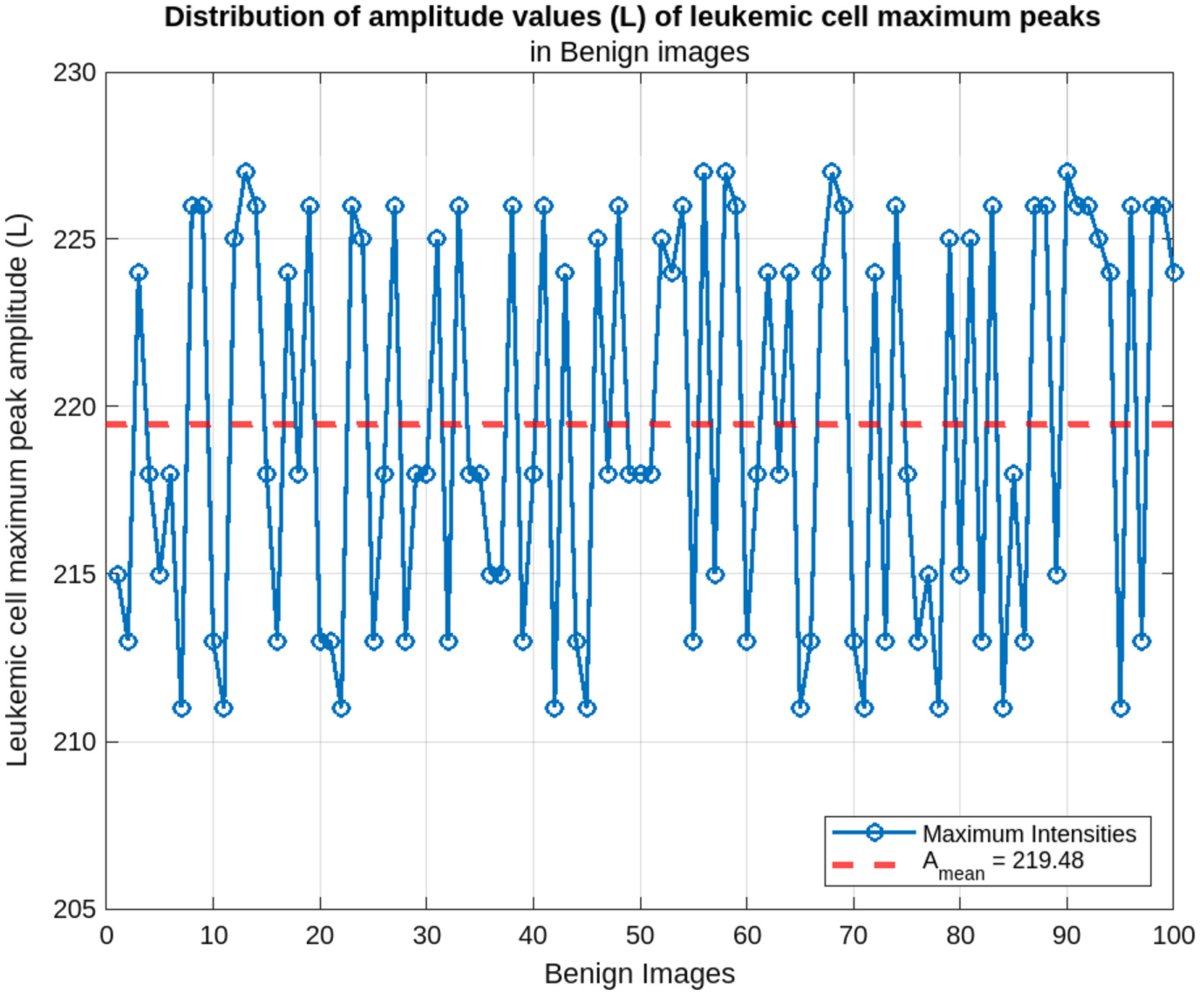
Benign cells maximum peak amplitude distribution.

Figure 8 shows the distribution of maximum amplitudes of leukemic cells in each input image of the benign class. Benign cells showed the highest maximum amplitudes with a mean of $A_{\text{mean}}=219.48$ and a range of [211; 227]. This distribution indicates a certain homogeneity of the benign cells and suggests a relatively intact cell structure with little alteration. These results confirm the integrity of the benign cells, with a high density and coherent composition.

**Fig. 9:**
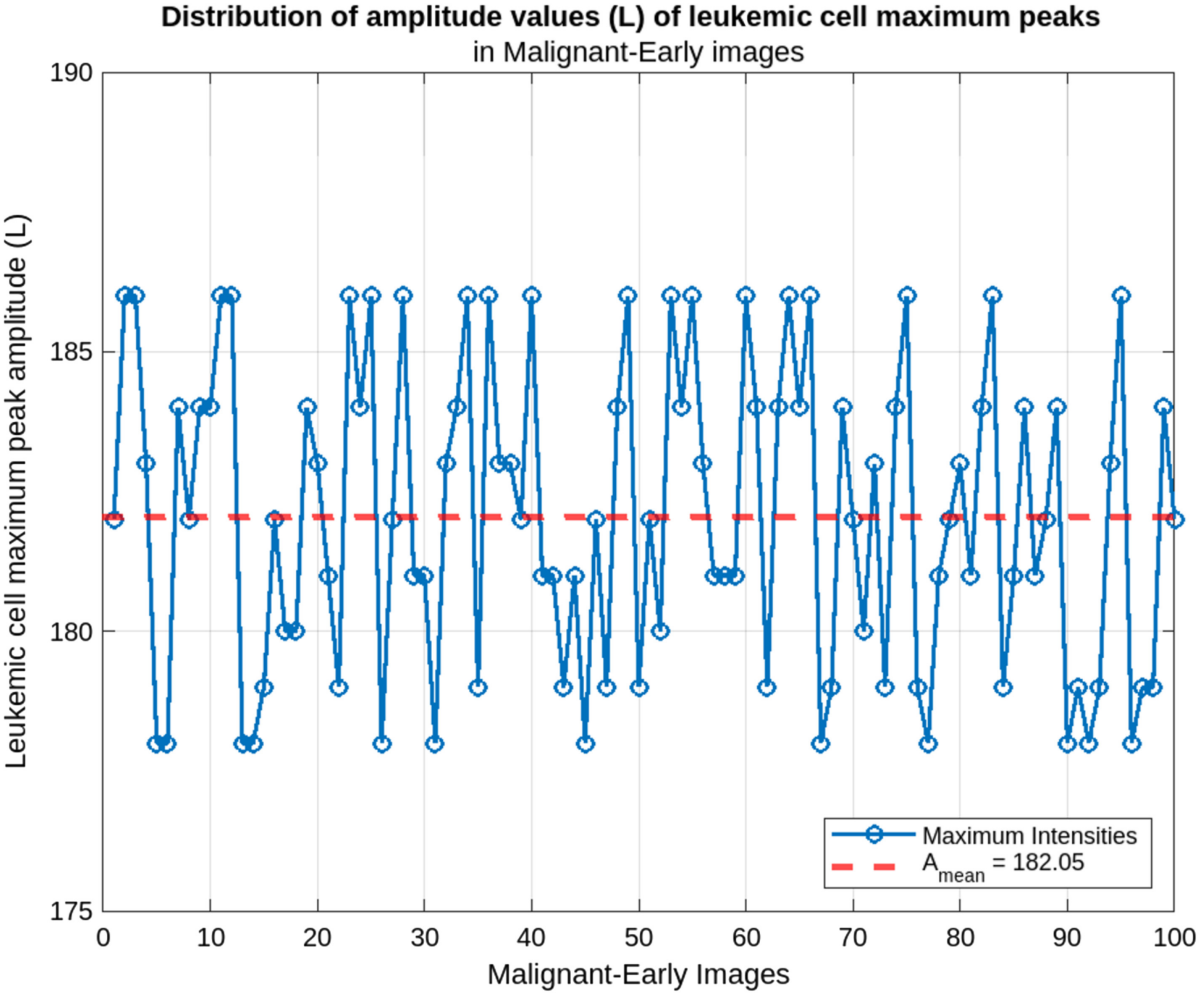
Malignant-early cells maximum peak amplitude distribution.

Figure 9 shows the distribution of maximum amplitudes for early-stage malignant class leukemic cells. The maximum amplitudes observed in this category are significantly lower than those of benign cells, with a mean of $A_{\text{mean}}=182.05$ and a range of variation [178; 186]. These values reflect the initial alterations occurring in the cells at this stage of the disease. The relative homogeneity of these amplitudes within this range reflects a still limited leukemic progression.

**Fig. 10:**
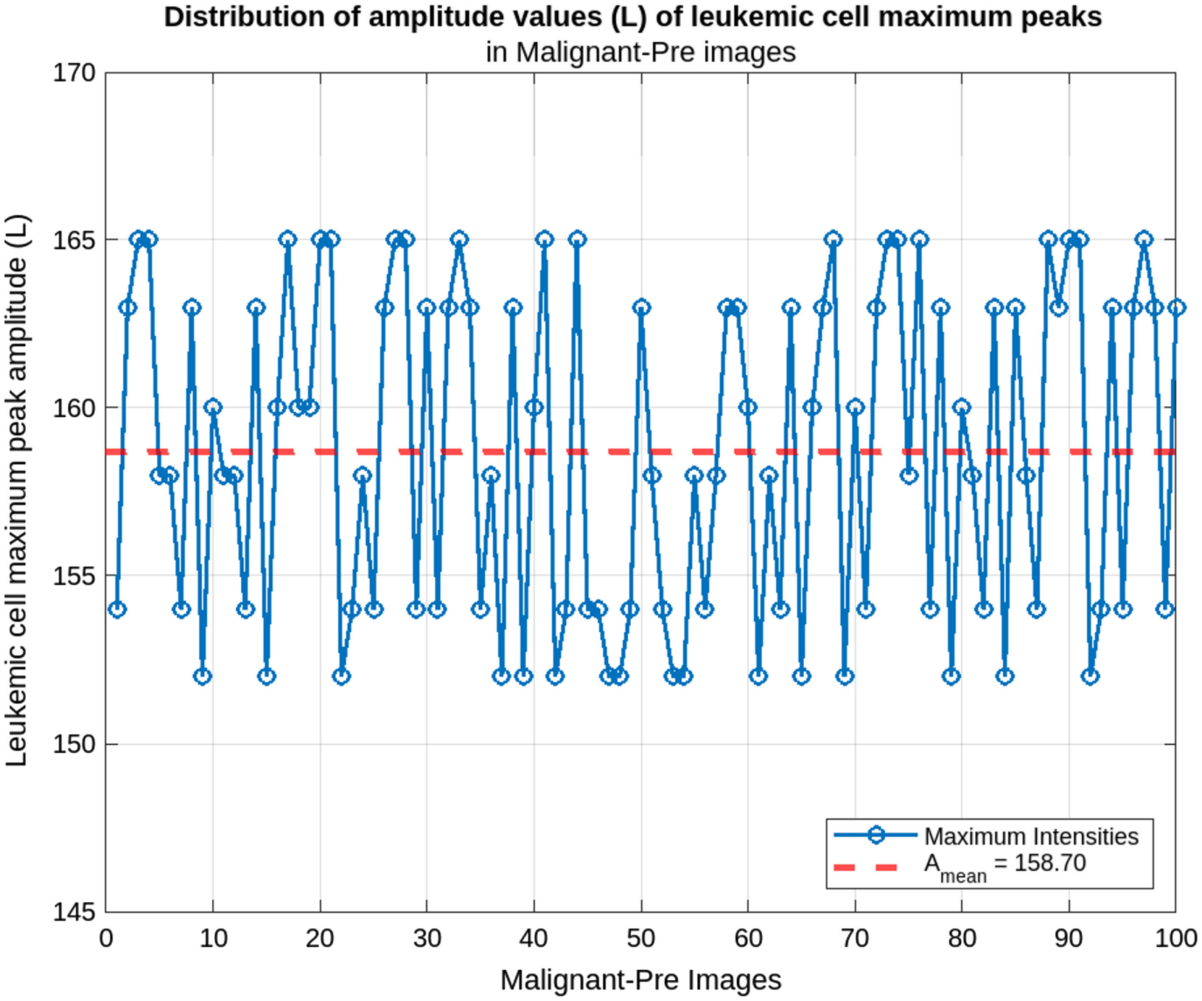
Malignant-pre cells maximum peak amplitude distribution.

The distribution of maximum amplitudes of leukemic cells in the pre-advanced stage is shown in Fig. 10. At this stage, the maximum amplitudes show a marked decrease, with a mean of $A_{\text{mean}}=158.70$ and a range of [152; 165]. This reduction reflects more pronounced cellular changes characteristic of disease progression. The widening of the range compared with the early stage indicates increasing cellular heterogeneity at this stage.

**Fig. 11:**
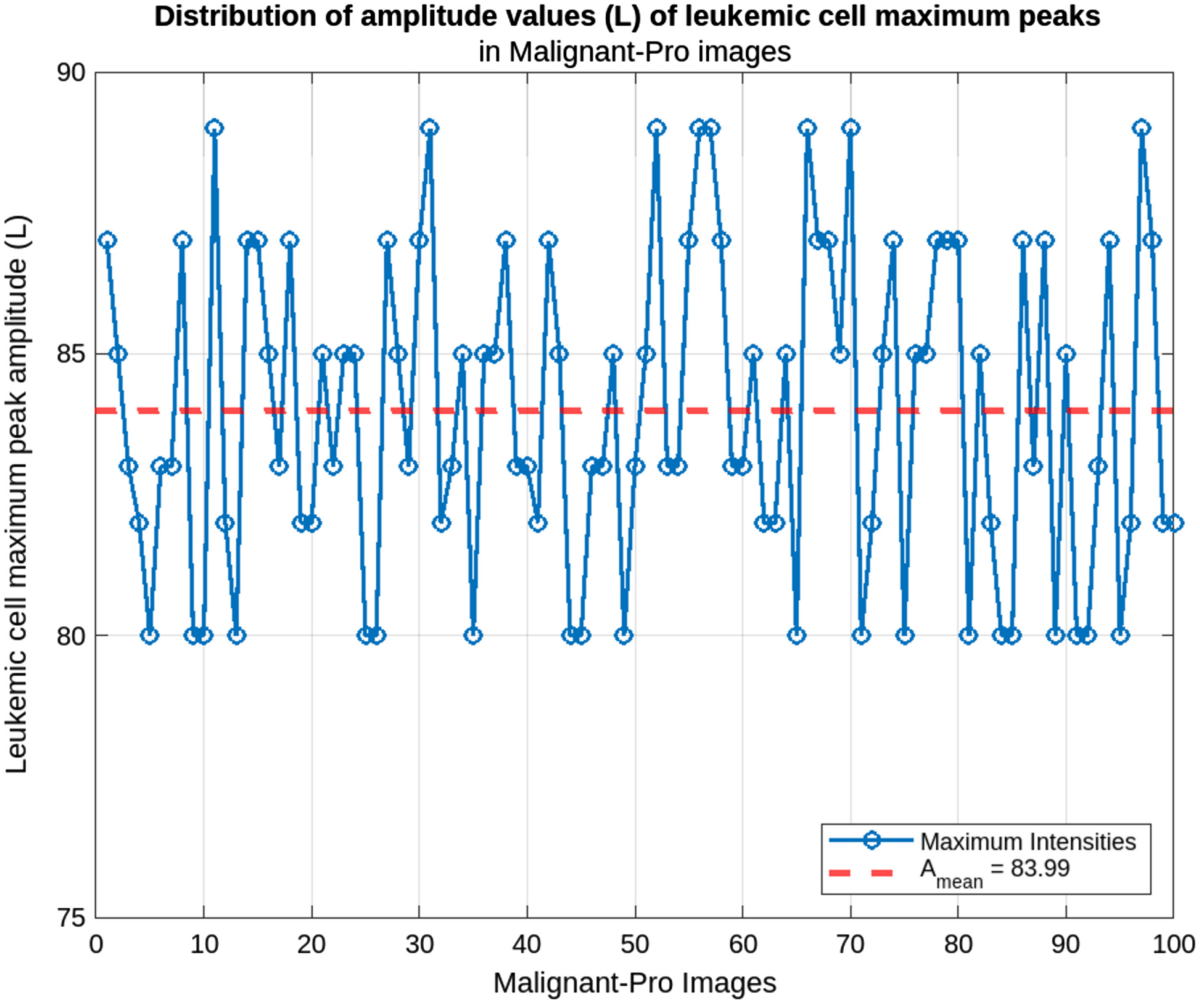
Malignant-pro cells maximum peak amplitude distribution.

Finally, Fig. 11 shows the distribution of maximum amplitudes of leukemic cells in the progressive stage. The lowest maximum amplitudes were observed in this class, with a mean of $A_{\text{mean}}=83.99$ and a range [80;89]. These values reflect advanced degradation of the cell structure, typical of the final stages of leukemic progression. The low variation in this range reflects increased uniformity of cell structural abnormalities.

These analyses show a progressive decrease in maximum amplitudes as the tumor progresses. Benign cells are characterized by high amplitudes, whereas malignant cells show progressively decreasing amplitudes, reflecting worsening cellular changes. This classification based on maximum amplitudes is a useful tool for differentiating between different types of leukemic cells and for monitoring their evolution.

Holographic reconstruction is used to show the fine details of leukemic cells. The use of the 2D Fourier transform increases contrast and makes it easier to distinguish among the different cells. In our case, we used the pure phase filter in the frequency domain, which allows us to exploit only the phase of the light wave without modifying its amplitude. This approach highlights the fine optical variations, such as differences in thickness and refractive index, specific to each cell type, and thus improves the contrast among the different classes (benign, malignant-early, malignant-pre, malignant-pro). Filtering acts as a high-pass filter, enhancing cell contours and facilitating the detection of internal structures essential for accurate segmentation.

To ensure isotropic treatment of spatial frequencies, we use a circular filter window centered on the Fourier plane with a radius between 10% and 20% of the Nyquist frequency. This configuration eliminates unwanted low-frequency components while preserving fine morphological details. In addition, we perform background subtraction on the phase maps reconstructed from a cell-free reference image to remove static interference and illumination inhomogeneities. This step significantly improves the visibility of cell structures, thereby optimizing the quality of the initial contours used for optical detection and segmentation. After reconstruction, the binary image obtained is used to detect the initial contour inside the cells using the OSH technique. This initial detection makes it possible to automate tumor segmentation using the active contour method, which is a semi-automatic method requiring manual selection of the initial contour.

**Fig. 12:**
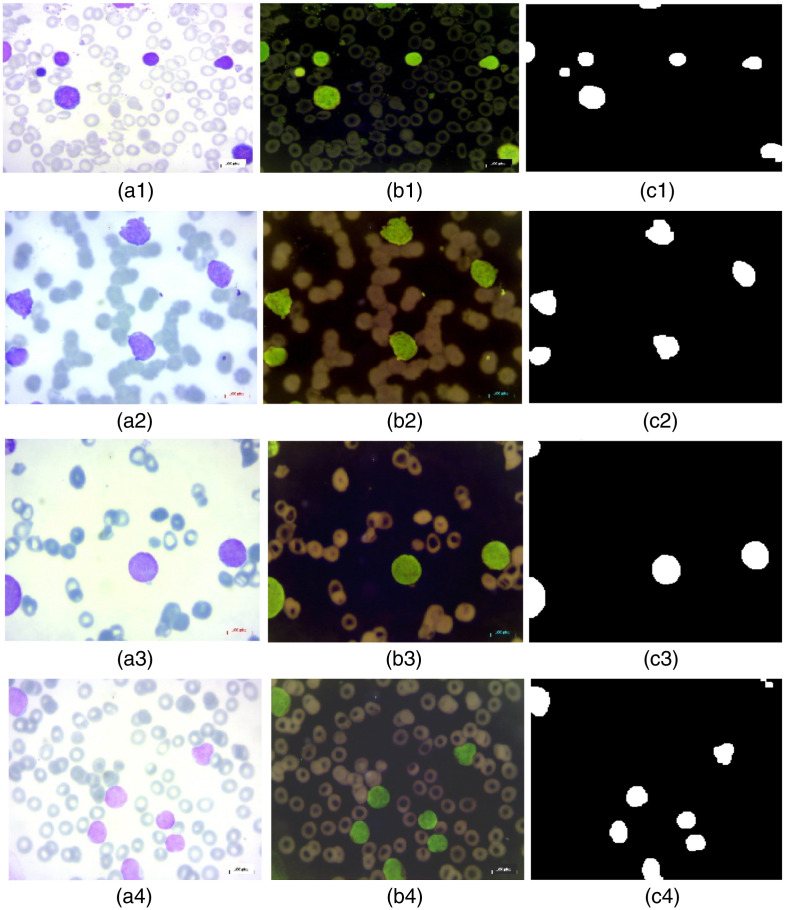
Holographic reconstruction of leukemic cells: (a1) input benign image, (b1) inverted benign image, (c1) reconstructed benign image, (a2) input malignant-early image, (b2) inverted malignant-early image, (c2) reconstructed malignant-early image, (a3) input malignant-pre image, (b3) inverted malignant-pre image, (c3) reconstructed malignant-pre image, (a4) input malignant-pro image, (b4) inverted malignant-pro image, and (c4) reconstructed malignant-pro image.

Figure 12 shows the resulting images after application of the Fourier transform and its inverse. The fine details of the cells are visible, making them easier to detect and segment.

In panel (a), the cells colored in dark purple are WBCs, and those in the background are RBCs. In panel (b), the images are inverted to improve the visibility of cell structures. Unlike thresholding or methods such as Cellpose/StarDist or other advanced segmentation techniques based on deep learning, which require a large volume of annotated data and can be sensitive to variations in contrast and lighting, the OSH method exploits the phase of the image, allowing more accurate segmentation that is independent of variations in intensity.

The use of active contours, particularly with the Chan-Vese method, allows cell segmentation to be refined. The contours are adjusted to fit exactly around the edges of the cells, even in areas where they are slightly overlapping or poorly defined. This step improves the accuracy of the segmentation, allowing leukemic cells to be better distinguished.

**Fig. 13:**
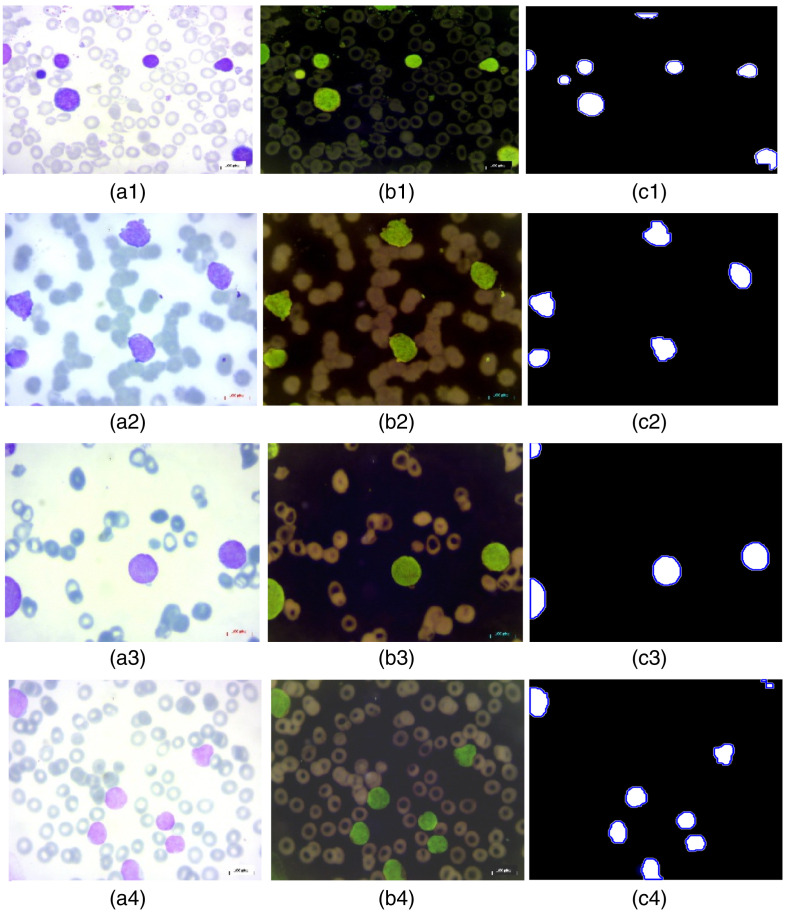
Cell segmentation after active contour application: (a1) input benign image, (b1) inverted benign image, (c1) cell segmentation after Chan-Vese application to benign image, (a2) input malignant-early image, (b2) inverted malignant-early image, (c2) cells segmentation after Chan-Vese application to malignant-early image, (a3) input malignant-pre image, (b3) inverted malignant-pre image, (c3) cell segmentation after Chan-Vese application to malignant-pre image, (a4) input malignant-pro image, (b4) inverted malignant-pro image, and (c4) cells segmentation after Chan-Vese application to malignant-pro image.

As demonstrated in Fig. 13, the application of active contours to segmented cells (illustrated in blue) enables the precise adjustment of their edges, thereby highlighting the method’s capacity to differentiate even small cells. In the case of leukemic cells, contour adjustment using active contours is less visible than in other complex tumor types, as demonstrated in our previous work on the detection and segmentation of brain tumors.^24^

For tumor segmentation, the hybrid method is evaluated using four similarity measures: sensitivity (sen), Dice coefficient (D), Hausdorff distance ($H_{d}$), and specificity (Spe), the values of which are given in Table 2. These measures are calculated as follows:

$$0\leq \mathrm{Sen}=\frac{\mathrm{TP}}{\mathrm{TP}+\mathrm{FN}}\leq 1,$$ (11)

$$0\leq D=\frac{\mathrm{TP}}{\mathrm{TP}+\frac{\mathrm{FP}+\mathrm{FN}}{2}}\leq 1,$$ (12)

$$0\leq \mathrm{Sep}=\frac{\mathrm{TN}}{\mathrm{TN}+\mathrm{FP}}\leq 1,$$ (13)

$$H_{d}(G,S)=\max\{\underset{a\in G}{\max}\;\underset{b\in S}{\min}\|a-b\|,\underset{b\in S}{\max}\;\underset{a\in G}{\min}\|b-a\|\}.$$ (14)

- Sensitivity: is 0.99, meaning that 99% of positive cases have been correctly identified.
- Specificity: is 0.99, meaning that 99% of negative cases have been correctly rejected.
- Dice: A coefficient of 0.98 to 0.99 indicates a 98% to 99% similarity between the predicted segmentation and the ground truth.
- Hausdorff distance: very low (∼1 to 2 pixels) guarantees accurate segmentation with few contour errors.

**Table 2:** Sensitivity, Dice, Hausdorff distance, and specificity obtained from the optimal contour of the blood-cell-cancer-(ALL)-4 class dataset images reached using our proposed method.

Metrics	Estimated values
Sensitivity [true positive rate (TPR)]	0.99 ± 0.0195
Dice similarity coefficient (DSC)	0.98 to 0.99 ± 0.0020
Hausdorff distance (HD)	1 to 2 ± 0.0992 pixels
Specificity [true negative rate (TNR)]	0.99 ± 0.0195

As shown in Table 3, a comparison is made of the performance of existing studies in terms of test accuracy and loss. This allows an assessment to be made of the effectiveness of the different approaches by highlighting their strengths and limitations. This comparative analysis provides an essential context for situating our method in relation to previous solutions.

**Table 3:** Test accuracy and loss of related work.

Methods	Algorithm/technique	Accuracy (%)	
		Inside tumor	Outside tumor
Automated blast cell detection for acute lymphoblastic leukemia diagnosis (2021)^36^	YOLOv4 for ALL_IDB1	92.00	08.00
	YOLOv4 for C_NMC-2019	96.00	04.00
Feature extraction of white blood cells using CMYK-moment localization and deep learning in acute myeloid leukemia blood smear microscopic image (2022)^37^	FCL	94.92	5.08
	RF	95.47	4.53
	SVM	96.41	3.59
	XGBoost	95.18	4.82
Hybrid inception v3 XGBoost model for acute lymphoblastic leukemia classification (2021)^38^	AlexNet	89.40	10.60
	DenseNet121	86.90	13.10
	ResNet18	91.70	8.30
	VGG16	92.40	7.65
	SqueezeNet	93.20	6.80
	MobileNetV2	95.80	4.20
Automated segmentation and classification of acute lymphoblastic leukemia blasts using OSH and AC methods	Proposed method	99.00	01.00

The proposed method combines innovative segmentation and automatic classification techniques, with in-depth analysis of the maximum values of cell amplitudes. These elements allow leukemic cells to be accurately and reliably segmented while ensuring high performance in terms of classification, including 99.00% accuracy and a low loss value of 1.00%, as shown in Table 3.

Furthermore, the proposed method shows a significant improvement in accuracy when compared with the deep-learning-based CNN model proposed by Hosseini et al., which uses a lightweight CNN model for cell classification on the same database. The aforementioned model achieves an accuracy of 94.30%, whereas the proposed method achieves an accuracy of 99.00%, which is a significant enhancement.

Unlike deep-learning-based approaches, our method provides a fast and robust solution for leukemic cell segmentation and classification while ensuring rigorous results through thorough evaluation.

## 6 Conclusion

This study has demonstrated the effectiveness of the combined method of optical scanning holography and active contours for the segmentation of leukemic blasts. The main results show that the integration of these innovative techniques, combined with a careful analysis of the maximum values of the cell amplitudes, allows leukemic cells to be segmented extremely accurately and reliably. The method performed exceptionally well, with a classification accuracy of 99.00%. These results highlight the significant advantages of holography and active contours in the prediction and monitoring of leukemia, paving the way for potential clinical applications to improve patient diagnosis and monitoring.

### 6.1 Future Perspective

Extending the approach of combining holography and active contours to the classification and analysis of other types of cancer cells, particularly those in solid tumors, offers considerable potential for the development of universal diagnostic tools. This could lead to improved early detection, monitoring, and personalized treatment of various cancers.

## Data Availability

The dataset used in this study, containing four classes of leukemic cells (benign, WBC malignant-early, WBC malignant-pre, and WBC malignant-pro) with 100 images per class, is publicly available on Figshare at: https://doi.org/10.6084/m9.figshare.29150819.v1 The MATLAB source codes developed for image processing are accessible at: https://doi.org/10.6084/m9.figshare.29150876 Figures illustrating the results presented in this paper can be found at: https://doi.org/10.6084/m9.figshare.29150885
